# Clinical applications of MRI-based artificial intelligence in spinal metastases: A systematic review

**DOI:** 10.1016/j.jbo.2026.100782

**Published:** 2026-07-06

**Authors:** Chunhua Hou, Anqi Wang, Jianru Xiao, Xiang Wang, Wei Xu

**Affiliations:** a University of Shanghai for Science and Technology, Shanghai 200093, China; b Department of Orthopedic Oncology, Shanghai Changzheng Hospital, Naval Medical University, Shanghai 200003, China

**Keywords:** Spinal metastases, Magnetic resonance imaging, Artificial intelligence, Deep learning, Radiomics, Systematic review

## Abstract

**Objective:**

This review systematically evaluates the current research landscape, methodological characteristics, and translational challenges of artificial intelligence (AI) integrated with magnetic resonance imaging (MRI) across the diagnostic and therapeutic pathway of spinal metastases, with the aim of informing clinical practice and future research.

**Methods:**

Following the Preferred Reporting Items for Systematic Reviews and Meta-Analyses (PRISMA) guidelines, we conducted a systematic search of PubMed, Web of Science, and the Cochrane Library. Original studies investigating AI models, including machine learning, deep learning, and large language models, developed from MRI data for spinal metastases were included. Two reviewers independently screened studies and extracted data. Sixty-one studies were included in the qualitative synthesis.

**Results:**

The included studies focused on four core clinical tasks: diagnosis and pathological classification (23 studies), clinical prognosis and risk stratification (17 studies), lesion detection and segmentation (16 studies), and automated clinical scoring and report analysis (5 studies). AI models showed promising performance across these tasks, with the highest area under the curve (AUC) for benign-malignant differentiation reaching 0.98 and the highest Dice similarity coefficient (DSC) for automatic lesion segmentation exceeding 0.85. Nevertheless, important limitations remain. Most studies were single-center retrospective investigations (73%), and the majority addressed isolated tasks rather than integrated clinical workflows. Important gaps also persist in multicenter generalizability, long-term survival prediction, and multimodal data integration.

**Conclusion:**

MRI-based AI has substantial potential to improve the diagnosis and management of spinal metastases. Future studies should emphasize large-scale, multi-center prospective validation and integrated intelligent systems supporting screening, decision-making, treatment response assessment, and long-term follow-up.

## Highlights

• MRI-based AI applications in spinal metastases were systematically mapped.
• MRI-AI supports diagnosis, segmentation, scoring, and risk prediction.
• AI tools may improve lesion assessment and radiotherapy workflows.
• Evidence for survival prediction and treatment selection remains limited.
• Multicenter prospective validation is needed before clinical translation.

## Graphical abstract

**Figure:**
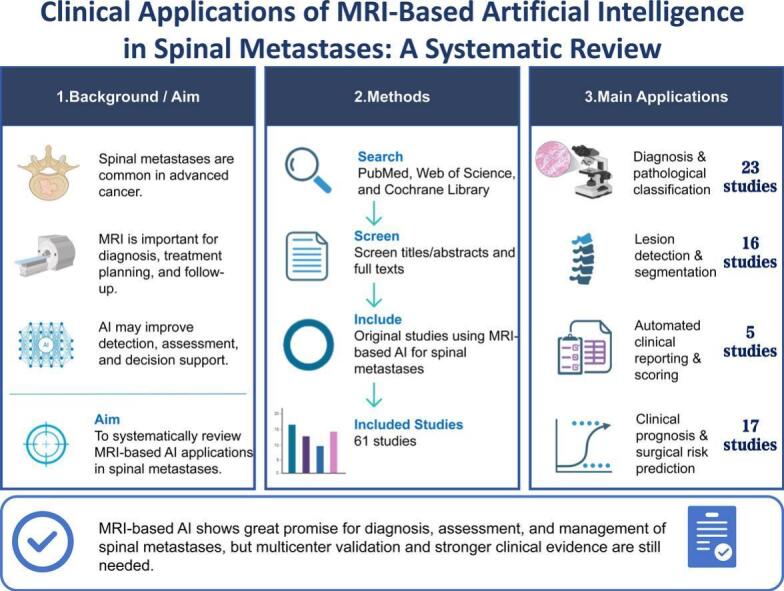

## 1 Introduction

One of the typical patterns of metastasis for patients with advanced malignancy is bone metastasis, and among the axial skeleton, the spine is frequently one of the most affected sites [1]. When a tumor grows in a vertebra, it can cause severe, unalleviated pain, pathological fracture and spinal instability, as well as metastatic epidural spinal cord compression (MESCC). Spinal cord or cauda equina compression may result in lower limb weakness, difficulty walking, urinary and bowel dysfunction, and even permanent paralysis of the legs [2]. Therefore, to reduce the risk of serious complications [3], prompt identification of early-stage spinal metastasis and timely assessment of neurological compression and instability are required.

Treatment decision-making for spinal metastases usually requires multidisciplinary collaboration. The Neurologic, Oncologic, Mechanical and Systemic (NOMS) framework indicates that clinical decision-making should take into account neurological compression, tumor sensitivity to treatment, mechanical stability of the spine and the patient's systemic condition simultaneously [4]. Therefore, the images need to show whether there is metastasis, and at the same time determine if MESCC is present, whether the spine is stable, and whether the patient is more suitable for surgery, radiotherapy or systemic therapy. Accuracy and consistency of the images will affect the following treatment.

X-ray, computed tomography (CT) and bone scintigraphy are still frequently employed in the diagnosis of spinal metastases to examine bone destruction, pathological fractures and other systemic bone metastases. However, early spinal metastatic lesions are frequently located in the bone marrow cavity. Before the onset of obvious cortical destruction or an osteoblastic reaction, these lesions may not be evident on X-rays or CT scans [5]. CT is useful for assessing cortical destruction and fractures, but it has relatively low sensitivity for the early detection of bone marrow infiltration, epidural soft tissue invasion, and the extent of spinal cord compression [6].

Magnetic resonance imaging (MRI) has excellent soft-tissue contrast and multi-sequence imaging capability, so it is now one of the main ways to diagnose and assess spinal metastases [7]. MRI is sensitive to early bone marrow infiltration and can show vertebral marrow involvement, epidural soft tissue invasion, intraspinal tumor extension, and spinal cord compression. MRI has a high detection rate for spinal metastatic lesions, and both the sensitivity and specificity at the lesion level are over 90% [8]. T1-weighted imaging (T1WI), T2-weighted imaging (T2WI), fat-suppressed sequences, and short tau inversion recovery (STIR) can reflect marrow signal changes and soft tissue invasion from different perspectives. Therefore, MRI is not only used for diagnosis, but is also commonly applied in MESCC grading, radiotherapy target delineation, preoperative evaluation, and post-treatment follow-up [9], [10].

However, the increasing amount of information provided by MRI also increases the workload of image interpretation and assessment. With prolonged survival of cancer patients, increased follow-up examinations, and wider use of whole-spine MRI and whole-body MRI, radiologists need to process a substantially larger number of images [11]. Patients with spinal metastases often have multiple lesions, with marked heterogeneity in lesion size, signal intensity, enhancement pattern, and anatomical location. Small lesions and lesions in complex anatomical regions may be easily overlooked. In addition, lesion segmentation, quantification of bone metastatic burden, and delineation of the spinal cord and organs at risk (OARs) are time-consuming tasks and may be affected by the reader's experience and fatigue [11], [12].

In recent years, deep learning (DL) has developed rapidly in medical image analysis. Convolutional neural networks (CNNs) and other models have been widely applied to image classification, lesion detection, image segmentation and prognostic prediction [13]. Artificial intelligence (AI) models can automatically extract high-dimensional features from images and are thus more suitable for processing multi-sequence and high-dimensional MRI data than traditional manual image interpretation or hand-crafted feature analysis. In the field of spinal metastases, AI has been explored for lesion detection and segmentation, benign-malignant differentiation, primary tumor prediction, radiotherapy-related image processing, treatment decision support, and prognostic evaluation [12].

To enhance readability for readers who are not familiar with MRI-based AI methods, the main technical terms, abbreviations, AI models and clinical terms used in this review are listed in Supplementary Table S1.

## 2 Methods

We employed the search strategy (((((MRI) OR (MR)) OR (magnetic resonance imaging)) AND (((deep learning) OR (machine learning)) OR (artificial intelligence))) AND (metasta*)) AND (((spine) OR (spinal)) OR (bone)) to systematically search PubMed, Web of Science and the Cochrane Library for related studies. No additional limits were set for the search scope. The inclusion criteria were original research papers on spinal metastases that used machine learning (ML), deep learning, or other artificial intelligence techniques and built models based on MRI data.

The exclusion criteria were as follows: (1) studies not related to bone metastasis or studies that focused only on primary tumors; (2) studies that did not use MRI-related information for modeling; (3) studies that did not use ML, DL or other AI methods; and (4) animal studies, reviews, conference abstracts, comments, or other non-original studies.

The titles and abstracts of all studies were first screened for relevance, and then the full texts of potentially eligible studies were examined. Two reviewers (WX and HCH) independently performed literature screening, eligibility assessment and data extraction. Any disagreements were resolved through discussion with a third evaluator (WAQ). This systematic review followed the recommended reporting items in Preferred Reporting Items for Systematic Reviews and Meta-Analyses (PRISMA) guidelines. The primary extracted information included study design, task type, model construction method, sample size and model performance indicators. Definitions of the main evaluation metrics are provided in Supplementary Table S2. Differences in the extracted data were reconciled through discussion.

## 3 Results

### 3.1 Search results

**Fig. 1:**
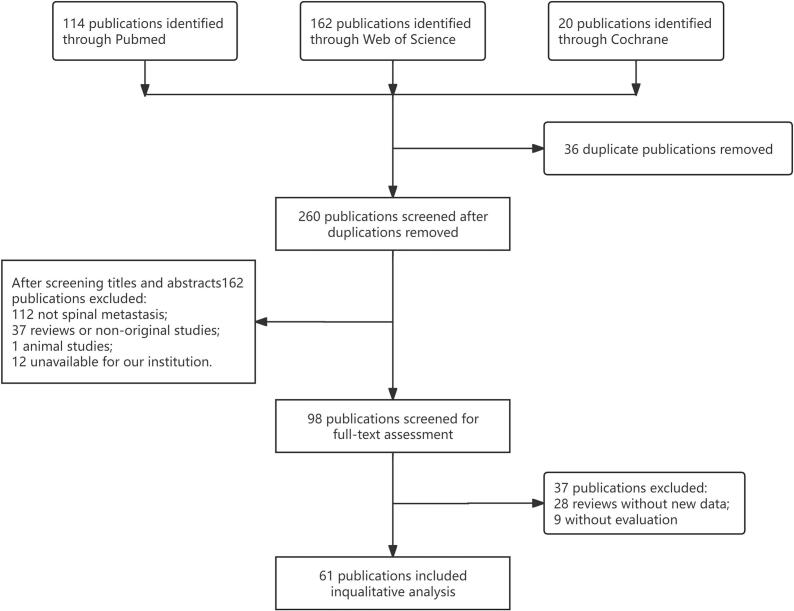
Flowchart of study selection for the systematic review of MRI-based artificial intelligence studies on spinal metastases.

A total of 296 related papers were first collected. Of these, 198 were excluded after screening the titles and abstracts. The remaining 98 articles were assessed for full-text eligibility, and 61 studies met the inclusion criteria and were finally included in this systematic review (Fig. 1). Based on the task types and performance indicators, the studies included in this work can be classified into four categories: diagnosis and pathological classification, automatic detection and precise segmentation, automated clinical reporting and scoring, and clinical prognosis and surgical risk prediction.

### 3.2 Study characteristics

**Fig. 2:**
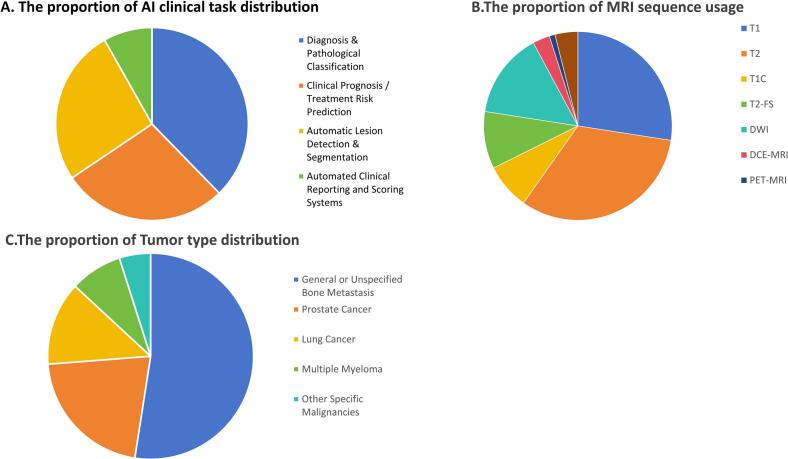
Of the included studies, the distribution of clinical tasks (A), MRI sequence usage (B), and tumor types in AI-based research on spinal metastases (C).

Among the 61 included studies, diagnosis and pathological classification were the most frequent task types, with 23 studies. Clinical prognosis and surgical risk prediction were studied in 17 papers, lesion automatic detection and precise segmentation in 16 papers, and automated clinical reporting and scoring system development in 5 papers (Fig. 2A).

Different MRI sequences were used in the included studies. Because some studies used multiple MRI sequences at the same time, sequence counts overlapped across studies. The three most frequent types of sequences were T2WI (33 studies), T1WI (28 studies) and diffusion-weighted imaging (DWI) (15 studies). Some studies also used functional sequences, such as dynamic contrast-enhanced MRI (DCE-MRI) and STIR (Fig. 2B).

Analysis of the study populations showed that about 52% of the studies, namely 32 studies, focused on general or unspecified bone metastatic disease and mainly aimed to develop broadly applicable detection, diagnostic or assessment methods. The other 29 studies were all on specific types of cancer. Among the single-cancer studies, prostate cancer occurred more often than others, with a total of 13 studies; among these 13, 11 were isolated prostate cancer studies and only 2 were combined studies. Lung cancer followed, and among the 8 studies, 7 were only on lung cancer and 1 was combined. Five studies reported multiple myeloma (Fig. 2C).

Among the study centers, most were single-center studies, making up about 73% of the total. There were 11 two-center studies and 5 multi-center studies. Among the three types of models, 32 were based on deep learning and 29 were based on machine learning.

### 3.3 Diagnosis and pathological classification: From visual recognition to virtual biopsy

This was the most studied part. The 23 studies in this category were mainly divided into the following four core tasks. These studies tried to extract MRI information that could not be obtained by the naked eye to help doctors evaluate the biological characteristics, primary sites and molecular signatures of lesions and tumors (Table 1).

**Table 1:** Applications of artificial intelligence integrated with MRI for diagnosis, differential diagnosis, pathological classification, and molecular prediction of spinal metastases.

Year	Title	Detected Modality	Detected Cancer	Classification Task	Sample Size	Center	AUC	ACC	DSC
2022	Automated Differentiation Between Osteoporotic Vertebral Fracture and Malignant Vertebral Fracture on MRI Using a Deep Convolutional Neural Network [14]	T1, T2	Mixed	Benign vs malignant vertebral fracture classification	97	1	0.984	0.96	
2024	The Classification of Metastatic Spine Cancer and Spinal Compression Fractures by Using CNN and SVM Techniques [15]	T1	Mixed	Compression fracture classification	248	1	0.9	0.98	
2023	Accurate Differentiation of Spinal Tuberculosis and Spinal Metastases Using MR-Based Deep Learning Algorithms [16]	T2	Mixed	Tuberculosis vs metastasis classification	121	4	0.98	0.98	
2022	Benign and Malignant Diagnosis of Spinal Tumors Based on Deep Learning and Weighted Fusion Framework on MRI [17]	T1, T2	Mixed	Benign vs malignant tumor classification	585	1	0.821		
2025	Deep Learning-Based Differentiation of Vertebral Body Lesions on Magnetic Resonance Imaging [18]	T1, T2	Mixed	Vertebral lesion classification	235/54	2		0.84	
2021	Radiomic Machine Learning Classifiers in Spine Bone Tumors: A Multi-Software, Multi-Scanner Study [19]	Routine MRI	Mixed	Bone tumor classification	181	1		0.94	
2022	Diffusion-Weighted MRI Radiomics of Spine Bone Tumors: Feature Stability and Machine Learning-Based Classification Performance [20]	DWI/ADC, T2	Mixed	Benign vs malignant classification	101	1	0.78	0.76	
2018	A Texture Analysis Approach for Spine Metastasis Classification in T1 and T2 MRI [21]	T1, T2	Mixed	Spinal Metastasis Classification	153	1		0.90	
2021	Deep Learning on MRI Images for Diagnosis of Lung Cancer Spinal Bone Metastasis [22]	T2, T2-FS	Lung	Spinal Metastasis Diagnosis	87	1		0.85	
2025	Deep Learning and Transformer-Based Feature Fusion of Conventional MRI for Differentiating Spinal Osteolytic Bone Metastases and Multiple Myeloma [23]	T1, T2, T2-FS	Myeloma	Myeloma vs metastasis classification	421/242	2	0.783	0.723	
2024	Radiomics Model Based on MRI to Differentiate Spinal Multiple Myeloma from Metastases: A Two-Center Study [24]	T1C, T2	Myeloma	Myeloma vs metastasis classification	210/53	2	0.87	0.86	
2022	Differentiation Between Spinal Multiple Myeloma and Metastases Originated from Lung Using Multi-View Attention-Guided Network [25]	T1C	Lung/Myeloma	Myeloma vs Lung-derived Metastasis Classification	217	1	0.77	0.79	
2019	A Triple-Classification Radiomics Model for the Differentiation of Primary Chordoma, Giant Cell Tumor, and Metastatic Tumor of Sacrum Based on T2-Weighted and Contrast-Enhanced T1-Weighted MRI [26]	T1C, T2	Mixed	Multi-class Sacral Tumor Classification	120	1	0.773	0.711	
2023	Contrast-Enhanced Magnetic Resonance Image Segmentation Based on Improved U-Net and Inception-ResNet in the Diagnosis of Spinal Metastases [27]	T1, T2, T2-FS	Mixed	Benign vs Metastatic Lesion Classification	81	1		0.98	
2025	Development and Validation of a Multi-Modal MRI-Based Deep Learning Framework for Differentiation of Intraspinal Tumors (ISMF-Net) [28]	T1, T2	General/Mixed	Intraspinal Tumor Classification	723/281	3		0.859	
2019	Differentiation of Spinal Metastases Originated from Lung and Other Cancers Using Radiomics and Deep Learning Based on DCE-MRI [29]	DCE	Lung	Primary Tumor Origin Prediction	61	1		0.79	
2023	Prediction of Primary Tumor Sites in Spinal Metastases Using a ResNet-50 Convolutional Neural Network Based on MRI [30]	T1, T2	Mixed	Primary Tumor Site Prediction	295	1	0.77	0.52	
2025	Identifying Primary Sites of Spinal Metastases: Expert-Derived Features vs. ResNet50 Model Using Nonenhanced MRI [31]	T1, T2, T2-FS	Mixed	Primary Tumor Origin Prediction	514	1	0.80	0.88	
2023	Identification of Origin for Spinal Metastases from MR Images: Comparison Between Radiomics and Deep Learning Methods [32]	T1C	Lung	Metastatic Origin Prediction	149/24	2	0.68	0.65	
2021	Multiparametric MRI-Based Radiomics Approaches for Preoperative Prediction of EGFR Mutation Status in Spinal Bone Metastases in Patients with Lung Adenocarcinoma [33]	T1, T2, T2-FS	Lung	EGFR Mutation Status Prediction	97	1	0.77		
2021	MRI-Based Radiomics Analysis for Predicting the EGFR Mutation Based on Thoracic Spinal Metastases in Lung Adenocarcinoma Patients [34]	T1, T2	Lung	EGFR mutation prediction	110/52	2	0.886		
2023	Comprehensive Analysis of Prediction of the EGFR Mutation and Subtypes Based on the Spinal Metastasis from Primary Lung Adenocarcinoma [35]	T1, T2-FS	Lung	EGFR Mutation and Subtype Prediction	257/42	2	0.82		
2022	Deep learning for preoperative prediction of the EGFR mutation and subtypes based on the MRI image of spinal metastasis from primary NSCLC [36]	Routine MRI	Lung	EGFR Mutation Prediction	257	1	0.76		

#### 3.3.1 Differentiation between metastatic and non-metastatic lesions

A total of 9 studies focused on distinguishing spinal metastases from non-metastatic lesions, mainly including the characterization of vertebral compression fractures, differentiation between spinal tuberculosis and metastasis, and multi-class classification of spinal lesions [14], [15], [16], [17], [18], [19], [20], [21], [22].

DL models based on conventional MRI showed good performance in the differentiation of vertebral compression fractures. Yoda et al. [14] built a CNN model to distinguish between osteoporotic vertebral fractures and malignant vertebral compression fractures. The T1WI-based model had an area under the curve (AUC) of 0.984 and an accuracy of 96.4%, and was as good as or better than spine surgeons. Another CNN/support vector machine (SVM) study showed that the sensitivity for identifying metastatic compression fractures could reach 1.000. Thus, it was proposed that deep MRI imaging features could provide additional support for fracture characterization [15].

Duan et al. [16] introduced DL models based on sagittal T2WI MRI for differentiating spinal tuberculosis from metastatic diseases. The best-performing model reached an accuracy of 98.7% in the internal validation set and 91.9% in the external test set, and had AUC values of 0.98 and 0.95, respectively. Therefore, the above results suggest that MRI-AI can help differentiate between infectious lesions and metastatic lesions when their images are too similar and the risk of misdiagnosis is high.

In addition, multi-sequence MRI fusion models and radiomics-based ML models have been used for multi-class differentiation among benign lesions, primary malignant tumors, and metastases [17], [18], [19], [20], [21], [22]. Multi-class problems are closer to the actual clinical situation and can better demonstrate the auxiliary value of a model in complex cases than simple binary classification.

In short, MRI-AI currently has relatively clear application scenarios for differentiating metastatic and non-metastatic spinal lesions, as well as for vertebral compression fractures, spinal tuberculosis, and multi-class spinal lesion classification. In addition to the above reasons, these extra sources of information can help reduce the subjectivity of image assessment.

#### 3.3.2 Differentiation between metastases and other malignant spinal Tumors

Six studies explored how to distinguish spinal metastases from other malignant spinal tumors, including multiple myeloma [23], [24], [25], sacral primary tumors [26], primary malignant spinal bone tumors [27], and intraspinal malignant tumors [28]. Instead of benign-malignant differentiation, these studies attempted to solve the problem of distinguishing between multiple types of malignancy in clinical practice, which is even more difficult.

For differentiating myeloma from metastasis, CNN-Transformer fusion models, MRI radiomics models, and multi-view attention networks all demonstrated some discriminative ability [23], [24], [25]. Among them, a two-center MRI radiomics model achieved an AUC of 0.870 and an accuracy of 0.862 [24]. A multi-view attention DL model based on contrast-enhanced T1WI (CE-T1WI) for distinguishing spinal myeloma from lung cancer spinal metastasis achieved an AUC of 0.7847 and an accuracy of 0.8108 [25]. Based on the above analysis, although myeloma and metastasis can both cause multiple destructive lesions in the vertebrae, differences in MRI signal intensity, texture, and lesion morphology may still be recognized by the model.

For special anatomical sites or complex tumor types, further explorations have also been conducted. A three-class radiomics model was employed to preoperatively classify lesions into sacral chordoma, giant cell tumor and metastasis, with an AUC of 0.773 and an accuracy of 0.711 [26]. A deep learning model for contrast-enhanced MRI was developed to differentiate spinal metastases from primary malignant spinal bone tumors; it demonstrated favorable performance, with an AUC of 0.9786 and an accuracy of 98.56% [27]. A multimodal MRI-based deep learning model was also employed to classify malignant intraspinal tumors, and among all these models, it yielded the highest overall mean AUC of 0.922 and an AUC of 0.845 for identifying spinal metastases [28].

Overall, MRI-AI has shown some promise for the further classification of malignant spinal lesions. Its value is not only to determine whether a lesion is malignant, but also to help clinicians differentiate among imaging-similar diseases, such as multiple myeloma, primary malignant spinal tumors, sacral tumors and malignant intraspinal tumors, and to provide supplementary evidence for subsequent pathological examination, surgical planning and selection of an integrated treatment strategy.

#### 3.3.3 Prediction of the primary tumor origin of bone metastases

Four studies developed models for identifying the primary origin of spinal metastases of unknown primary origin, mainly including binary differentiation of lung cancer-derived metastases and multi-class prediction among common primary tumors [29], [30], [31], [32].

For lung cancer-derived spinal metastases, one study used a convolutional long short-term memory (CLSTM) model based on DCE-MRI. The model analyzed signal changes during dynamic enhancement. It distinguished lung cancer-derived from non-lung cancer-derived spinal metastases, with an average accuracy of approximately 0.81 [29]. Another study based on preoperative CE-T1WI showed that the DL model achieved an accuracy of 0.72 and an AUC of 0.76 in the external test set, outperforming radiomics models and radiologist assessment [32]. Thus, tumor vascularity, enhancement patterns and other internal heterogeneities in contrast-enhanced MRI can be used to distinguish lung metastases from other tumors.

A ResNet-50 residual network model based on non-contrast MRI for multiple cancer primary tumor classification of spinal metastases has been developed to distinguish among lung cancer, kidney cancer, breast cancer, thyroid cancer, and prostate cancer. In the five-class task, the top-1 accuracy and AUC-ROC of the model were 52.97% and 0.77, respectively. When the number of classes was reduced to three, the top-1 accuracy reached 67.16% and the AUC-ROC rose to 0.85 [30]. Another study further compared an ML model based on expert image features with a ResNet50 DL model. Based on the above results, the top-3 accuracy of the ML model based on expert image features reached 0.88 and its AUC was 0.80, exceeding that of ResNet50 [31].

MRI-AI can provide supplementary information to the first assessment and help narrow down the source area of spinal metastases when the primary cause of metastasis is unknown. Contrast-enhanced MRI and DCE-MRI are more sensitive to changes in tumor blood supply and enhancement characteristics, but non-contrast MRI is more easily available. At present, these models are more suitable for narrowing down the search area of the primary tumor and proposing priority screening directions.

#### 3.3.4 Molecular subtype and gene mutation prediction

Four studies investigated non-invasive prediction of epidermal growth factor receptor (EGFR) mutation status and common sensitive mutation subtypes in patients with lung cancer spinal metastases. These studies sought to employ MRI imaging characteristics to aid in determining the molecular information of tumors and provided an initial exploration of imaging-based “virtual biopsies” [33], [34], [35], [36].

Early studies mainly used MRI radiomics. One study built a fusion radiomics model using T1WI, T2WI, and fat-suppressed T2-weighted imaging (T2-FS) to predict EGFR mutation status. The model achieved an AUC of 0.891 in the training set and 0.771 in the validation set [33]. Another study on thoracic vertebral metastases added smoking status to the radiomics features to build a clinical-radiomics nomogram, and it achieved an AUC of 0.821 in a time-independent validation set [34]. Based on the above results, texture, signal and other morphological features in multi-sequence MRI may be related to EGFR mutation status.

Later studies focused on EGFR-sensitive mutation subtypes. Cao et al. [35] built a clinical-radiomics nomogram using T1W and T2-FS sequences. In the external validation set, the AUCs for predicting overall EGFR mutation, exon 19 mutation, and exon 21 mutation were 0.780, 0.846, and 0.818, respectively. That is to say, MRI may be used to determine whether an EGFR mutation exists and, at the same time, to predict common sensitive mutation subtypes.

Some studies also used end-to-end deep learning models. The AUC values of the internal and external validation sets for the prediction of EGFR mutation status by the CM-EfNet model were 0.851 and 0.764, respectively. For distinguishing exon 19 from exon 21 mutations, the external validation AUC was 0.687 [36]. Gradient-weighted class activation mapping (Grad-CAM) heatmaps were used in this paper to show the areas of the image that the model focused on during prediction, providing some interpretability for the model's results.

Overall, MRI features of lung cancer spinal metastases may be related to EGFR mutation status and common sensitive mutation subtypes. These models are not intended to replace genetic tests but to provide non-invasive supplementary information for EGFR mutation risk stratification when tissue samples are unavailable, biopsy is difficult, or the results of genetic tests are pending.

### 3.4 Lesion detection, segmentation, and radiotherapy target delineation in spinal metastases

The 16 included studies can be broadly classified into two categories: automatic lesion detection and segmentation, and radiotherapy-related image processing and planning assistance. Based on the above data, artificial intelligence has shown promising results in improving the efficiency and consistency of MRI examination for spinal and bone metastases, and in providing additional support for lesion quantification, follow-up comparison, radiotherapy planning, etc. (Table 2).

**Table 2:** Applications of artificial intelligence integrated with MRI for lesion detection, segmentation, image processing, and radiotherapy planning in spinal metastases.

Year	Title	Detected Modality	Detected Cancer	Classification Task	Sample Size	Center	AUC	ACC	DSC
2024	Automated Detection and Segmentation of Bone Metastases on Spine MRI Using U-Net: A Multicenter Study [37]	T1, T1C	Mixed	Automated Lesion Detection and Segmentation	302	3			0.699
2023	Computer-aided diagnosis of skeletal metastases in multi-parametric whole-body MRI [38]	T1, T2, DWI	Mixed	Whole-body Metastasis Detection	30	1			0.53
2019	Segmentation of Vertebral Metastases in MRI Using an U-Net like Convolutional Neural Network [39]	T1, T2	Mixed	Vertebral Metastasis Segmentation	38	1			0.738
2017	A Multi-Resolution Approach for Spinal Metastasis Detection Using Deep Siamese Neural Networks [40]	T1, T2-FS	Mixed	Small Lesion Detection	26	1			
2023	Context-Aware Transformers for Spinal Cancer Detection and Radiological Grading [41]	T1, T2	Mixed	Spinal Tumor Detection and Radiological Grading	2295	6	0.80		
2021	Detection and Segmentation of Pelvic Bones Metastases in MRI Images for Patients With Prostate Cancer Based on Deep Learning [42]	DWI/ADC, T1	Prostate	Pelvic Bone Metastasis Detection and Segmentation	859	2	0.94		0.85
2024	Deep learning assisted atlas-based delineation of the skeleton from Whole-Body Diffusion Weighted MRI in patients with malignant bone disease [43]	DWI/ADC	Myeloma / Prostate	Skeletal Segmentation	55	1			0.743
2024	Deep Learning for Delineation of the Spinal Canal in Whole-Body Diffusion-Weighted Imaging: Normalising Inter- and Intra-Patient Intensity Signal in Multi-Centre Datasets [44]	DWI/ADC	Myeloma / Prostate	Spinal Canal Segmentation	137	5			0.87
2021	Fully automated pelvic bone segmentation in multiparameteric MRI using a 3D convolutional neural network [45]	DWI/ADC	Prostate	Organ-at-risk Segmentation	324	1			0.85
2019	Generation of PET Attenuation Map for Whole-Body Time-of-Flight 18F-FDG PET/MRI Using a Deep Neural Network Trained with Simultaneously Reconstructed Activity and Attenuation Maps [46]	PET/MRI	Mixed	CT Attenuation Map Generation	100	1			0.77
2022	Feasibility of accelerated whole-body diffusion-weighted imaging using a deep learning-based noise-reduction technique in patients with prostate cancer [47]	DWI/ADC	Prostate	Metastasis Diagnosis	17	1			
2023	Machine learning based gray-level co-occurrence matrix early warning system enables accurate detection of colorectal cancer pelvic bone metastases on MRI [48]	DWI/ADC	Colorectal cancer	Bone Metastasis Prediction	614	1	0.91		
2019	MR-based treatment planning in radiation therapy using a deep learning approach [49]	CT, T1	Mixed	MR-guided radiotherapy planning	50	1	–		0.85
2023	Deep learning-based magnetic resonance imaging of the spine in the diagnosis and physiological evaluation of spinal metastases [50]	T1, T2	Mixed	Spinal Metastasis Diagnostic Evaluation	941	1		0.96	
2021	Generating Virtual Short Tau Inversion Recovery (STIR) Images from T1- and T2-Weighted Images Using a Conditional Generative Adversarial Network in Spine Imaging [51]	T1, T2	Mixed	STIR Image Generation	753	1			
2023	MedFusionGAN: multimodal medical image fusion using an unsupervised deep generative adversarial network [52]	CT, T1C	Mixed	CT/MRI Image Fusion	230	1			

#### 3.4.1 Automatic screening and segmentation of full-field bone metastatic lesions

Eleven studies focused on automatic detection and segmentation of spinal and bone metastatic lesions, as well as MRI workflow optimization [[37], [38], [39], [40], [41], [42], [43], [47], [48], [50], [51]]. These studies primarily aimed to solve problems such as the large number of whole-spine or whole-body MRI images, the long time required for manual interpretation, the possibility of missing small lesions, and the difficulty of objectively comparing the extent of disease before and after treatment.

Kim et al. [37] built a U-Net model based on multi-center whole-spine MRI for the assessment of whole-spine lesions. In the external test set, the model had a Dice similarity coefficient (DSC) of 0.699 and a lesion detection sensitivity of 0.857; thus, it can be used for the rapid localization of suspicious metastatic lesions. Whole-body MRI-related studies have further focused on quantifying bone metastatic burden, and lesions were automatically segmented to calculate indicators such as tumor volume and apparent diffusion coefficient (ADC) before and after treatment for comparison in imaging studies [38].

To identify small lesions and those in complex anatomical areas, multi-resolution Siamese neural networks and Transformer models have been used to integrate multi-scale image patches, adjacent slice information, and multi-sequence information for more stable lesion recognition [[40], [41]]. Three-dimensional U-Net (3D U-Net) models have also been applied to detect and segment lesions in pelvic bone metastases to achieve good patient-level staging performance with external data [42]. For local lesion segmentation, a U-Net-like model based on T1WI achieved a mean DSC of 73.84% for vertebral metastasis delineation [39].

Some studies also aimed to improve the MRI imaging workflow. Haubold et al. [51] used a conditional generative adversarial network (cGAN) to generate STIR images from T1WI and T2WI. The virtual STIR images were close to real STIR images in most spinal lesion assessments. Tajima et al. [47] used deep learning denoising reconstruction for whole-body diffusion-weighted imaging with background body signal suppression (DWIBS). This method improved image quality and the visualization of bone metastatic lesions, while also shortening scan time.

Research on the automatic detection and segmentation of lesions has generally moved from the simple identification of lesions to the delineation of lesion extent, quantification of bone metastatic burden, and optimization of MRI image quality. For patients with multiple bone metastases or long-term follow-up, the above methods can help doctors find suspicious lesions earlier and offer objective imaging indices for comparison before and after treatment.

#### 3.4.2 Radiotherapy-related image processing and planning assistance

Five studies focused on radiotherapy-related image processing and planning assistance, including automatic normal structure segmentation, MRI signal standardization, pseudo-CT generation, bone structure correction, and CT/MRI image fusion [[44], [45], [46], [49], [52]].

A U-Net model based on whole-body diffusion-weighted imaging (WB-DWI) was used for normal structure segmentation to automatically delineate the spinal cord and surrounding cerebrospinal fluid regions; DSC, precision, and recall in the test set all exceeded 0.87 [44]. A 3D U-Net model for pelvic MRI was developed to segment bony structures in the lumbar spine, sacrococcyx, ilium, acetabulum and femoral head, with mean DSC values of about 0.80–0.85 [45]. These models may reduce the amount of manual delineation and provide a foundation for bone metastatic lesion localization and quantitative analysis.

For MRI-only radiotherapy planning, the deepMTP model generated pseudo-CT images from a single three-dimensional T1WI scan. The model achieved a bone tissue DSC of 0.85 and a mean absolute error of about 75 Hounsfield units (HU). Radiotherapy plans based on pseudo-CT showed only small differences from conventional CT-based plans [49]. Research on attenuation correction for positron emission tomography/magnetic resonance imaging (PET/MRI) has also shown that DL methods can improve the detection of bone structures and achieve a bone-region DSC of 0.77 [46]. In addition, CT/MRI fusion models can preserve both the bone-structure information in CT and the soft-tissue contrast of MRI to provide supplementary imaging for the delineation of targets and organs at risk [52].

Overall, radiotherapy-related studies mainly focus on image processing before radiotherapy. Their aim is not to directly detect metastatic lesions. Instead, they aim to use MRI images to obtain more information for structure delineation, bone structure recognition, dose calculation and treatment planning before radiotherapy, thus improving the efficiency of pre-radiotherapy image processing.

### 3.5 Automated clinical scoring and intelligent report parsing in spinal metastases

Five studies focused on automatic grading and report information extraction of imaging results in spinal metastases, mainly including Bilsky grading of MESCC, automatic calculation of the Spinal Instability Neoplastic Score (SINS), and bone metastasis report screening and referral assessment [53], [54], [55], [56], [57] (Table 3).

**Table 3:** Applications of artificial intelligence integrated with MRI for automated clinical scoring and report analysis in spinal metastases.

Year	Title	Detected Modality	Detected Cancer	Classification Task	Sample Size	Center	AUC	ACC	ICC
2025	Evaluating the Accuracy of Privacy-Preserving Large Language Models in Calculating the Spinal Instability Neoplastic Score (SINS) [54]	MRI report	Mixed	LLM-based SINS Evaluation	124	1	/	/	0.984
2025	Large Language Model (LLM)-Predicted and LLM-Assisted Calculation of the Spinal Instability Neoplastic Score (SINS) Improves Clinician Accuracy and Efficiency [55]	MRI report	Mixed	LLM-based SINS Evaluation	60	1	/	/	0.993
2025	In-context learning enables large language models to achieve human-level performance in spinal instability neoplastic score classification from synthetic CT and MRI reports [56]	MRI report	Mixed	LLM-based SINS Classification	100	1	/	0.96– 0.98	/
2024	Automated Spinal MRI Labelling from Reports Using a Large Language Model [57]	MRI report	Mixed	Automated MRI Report Labeling	56,924	1	1.000	1.000	/
2022	Deep Learning Model for Classifying Metastatic Epidural Spinal Cord Compression on MRI [53]	T2	Mixed	epidural spinal cord compression grading	247	1	/	/	/

For SINS-related studies, ICC or correct classification rate was reported instead of conventional accuracy. For automated report labeling, balanced accuracy was used.

Hallinan et al. [53] created a DL model to distinguish between low-grade and high-grade spinal cord compression in Bilsky grading. The sensitivity and specificity were 97.6% and 93.6% in the internal test set, and 89.9% and 98.1% in the external test set, respectively. This model can help clinicians quickly identify severe cases of compression.

For SINS scoring, large language models (LLMs) were used to assist score calculation and stability stratification. Chan et al. [54] found that Claude 3.5 showed a high degree of agreement with expert consensus in the calculation of total SINS, with an intraclass correlation coefficient (ICC) of 0.984 and a stability stratification accuracy of 98.4%. Another study found that LLM-assisted physician scoring also increased agreement, with an ICC of 0.993, and reduced scoring time [55]. When clear scoring rules and a few examples were provided, the accuracy of LLM-based stability classification reached 96%–98% [56].

Park et al. [57] employed a large language model for report screening to automatically extract data from spinal MRI reports, and generally classified whether spinal cancer or stenosis was mentioned into structured labels. The above way can reduce the amount of manual annotation and provide data for training a classification model for MRI images. Based on the results of this study, a Llama3-based method was employed for report label extraction and achieved an accuracy of 1.000 for spinal cancer identification.

In short, AI can assist clinical workflows including Bilsky grading, SINS scoring and radiology report analysis. Currently, these tools are better suited for clinical alerting, preliminary screening and automated scoring verification.

### 3.6 Metastasis risk prediction and treatment-related risk assessment

The 17 studies mentioned above generally focused on predicting the risk of primary cancer bone metastasis or distant metastasis, as well as treatment-related risk and response assessment for patients with spinal or bone metastases. These studies suggest that MRI-AI can provide auxiliary information for high-risk patient identification, pretreatment risk assessment and treatment response follow-up (Table 4).

**Table 4:** Applications of artificial intelligence integrated with MRI for metastasis prediction, risk stratification, and clinical outcome assessment.

Year	Title	Detected Modality	Detected Cancer	Classification Task	Sample Size	Center	AUC	ACC	DSC
2025	Enhancing bone metastasis prediction in prostate cancer using quantitative mpMRI features, ISUP grade and PSA density: a machine learning approach [58]	T1, T2, DWI, DCE	Prostate	Bone metastasis prediction	122	1	0.91	0.92	
2024	A semi-automatic deep learning model based on biparametric MRI scanning strategy to predict bone metastases in newly diagnosed prostate cancer patients [59]	T2, DWI	Prostate	Bone metastasis prediction	318/96	2	0.934	0.902	0.607
2023	A machine learning radiomics model based on bpMRI to predict bone metastasis in newly diagnosed prostate cancer patients [60]	DWI/ADC, T2	Prostate	Bone metastasis prediction	284/64	2	0.928	0.884	
2025	Machine learning-based identification of high-risk bone metastasis factors after radical prostatectomy in prostate cancer [61]	T2	Prostate	Bone metastasis risk prediction	1161	1	0.926	0.847	
2025	MRI Radiomics and Automated Habitat Analysis Enhance Machine Learning Prediction of Bone Metastasis and High-Grade Gleason Scores in Prostate Cancer [62]	T2	Prostate	Bone Metastasis and High-grade Gleason Score Prediction	214	1	0.90		0.97
2025	Predicting bone metastasis and high-grade Gleason scores in prostate cancer: a retrospective study integrating clinical features and magnetic resonance imaging radiomics [63]	T1, T2, T2-FS, DWI	Prostate	Bone metastasis prediction	168	1	0.875		
2024	Deep learning algorithm-based multimodal MRI radiomics and pathomics data improve prediction of bone metastases in primary prostate cancer [64]	DWI/ADC, T2	Prostate	Bone metastasis prediction	211	1	0.93		
2022	Prediction for Distant Metastasis of Breast Cancer Using Dynamic Contrast-Enhanced Magnetic Resonance Imaging Images under Deep Learning [65]	DCE	Breast	Distant Metastasis Prediction	96	1	0.76	0.80	
2020	Radiomics-based machine-learning method for prediction of distant metastasis from soft-tissue sarcomas [66]	T1, T2	Mixed	Distant Metastasis Prediction	77	1	0.902	0.913	
2023	Clinicomics-guided distant metastasis prediction in breast cancer via artificial intelligence [67]	DWI/ADC	Breast	Distant Metastasis Risk Prediction	186	1			
2020	Distant metastasis prediction via a multi-feature fusion model in breast cancer [68]	T2, T2-FS	Breast	Distant Metastasis Prediction	201	1	0.85	0.76	
2023	Automatic tumor segmentation and metachronous single-organ metastasis prediction of nasopharyngeal carcinoma patients based on multi-sequence magnetic resonance imaging [69]	T1, T2, T1C	Nasopharyngeal carcinoma	Metachronous Single-organ Metastasis Prediction	186	1	0.775		
2024	Prediction of bone invasion of oral squamous cell carcinoma using a magnetic resonance imaging-based machine learning model [70]	T1, T2	Oral cancer	Bone invasion prediction	86	1	0.99		
2026	Development of a preoperative prediction tool for massive intraoperative blood loss in spinal metastases surgery integrating mri and clinical data: a multicenter study [71]	T1C	Mixed	Massive Intraoperative Blood Loss Prediction	702	2	0.901		
2023	MRI feature-based radiomics models to predict treatment outcome after stereotactic body radiotherapy for spinal metastases [72]	T1, T2, T2-FS	Mixed	Treatment response prediction	194	1	0.828		
2021	Radiomic modeling to predict risk of vertebral compression fracture after stereotactic body radiation therapy for spinal metastases [73]	CT, T1	Mixed	vertebral compression fracture risk prediction	74	1	0.87		
2025	AI-driven software for automated quantification of skeletal metastases and treatment response evaluation using whole-body diffusion-weighted MRI (WB-DWI) in advanced prostate cancer [74]	WB-DWI / ADC	Prostate	Automatic Segmentation and Treatment Response Evaluation	171	multi-centre		0.805	0.6

#### 3.6.1 Noninvasive prediction of bone metastasis risk in primary cancers

Thirteen studies constructed risk prediction models for bone metastasis and distant metastasis based on primary tumor MRI in prostate cancer [58], [59], [60], [61], [62], [63], [64], breast cancer [[65], [67], [68]], nasopharyngeal carcinoma [69], soft tissue sarcoma [66], and oral squamous cell carcinoma [70]. Although these studies did not directly inspect bone metastatic lesions in MRI images, they intended to identify patients who were at high risk of developing future bone or distant metastases based on imaging features of the primary tumor.

Prostate cancer was the most studied cancer type. Several studies showed that radiomics, deep learning, and clinical fusion models based on biparametric or multiparametric MRI can be used to predict bone metastasis risk in newly diagnosed or postoperative patients [58], [59], [60], [61], [62], [63], [64]. Some models achieved AUCs close to 0.90 in external validation. This suggests that MRI features may add useful information to traditional clinical indicators, such as prostate-specific antigen (PSA) and International Society of Urological Pathology (ISUP) grade [59], [60].

Breast cancer studies mainly focused on distant metastasis risk prediction. Models that combined MRI features with clinicopathological factors performed better than models based only on imaging or clinical information [[65], [67], [68]]. Research on nasopharyngeal carcinoma also found that multi-sequence MRI-based models can predict the risk of post-treatment single-organ distant metastasis, such as bone metastasis [69].

Texture, signal and enhancement features of primary tumor MRI may indicate tumor heterogeneity and aggressiveness. Combining imaging features with clinicopathological data can help identify high-risk patients for bone or distant metastasis and provide auxiliary support for subsequent imaging examinations and follow-up planning.

#### 3.6.2 Perioperative and treatment-related risk prediction

The four studies focused on treatment-related risk and therapeutic response assessment for patients with spinal or bone metastases, including intraoperative prediction of massive blood loss, prediction of vertebral compression fracture after stereotactic body radiotherapy (SBRT), local progression after radiotherapy, and automatic quantification of whole-body bone metastatic burden [71], [73], [72], [74].

Wang et al. [71] developed a combined model based on preoperative CE-T1WI radiomics features and clinical factors to predict the risk of massive blood loss during surgery for spinal metastases in a study of surgical risk prediction. It achieved AUCs of 0.901 and 0.885 in the internal and external test sets, respectively. This performance was better than that of the clinical model alone. The model can be used to prepare blood before surgery, plan the operation and consider prophylactic embolization.

Gui et al. [73] predicted the risk of vertebral compression fractures after radiotherapy using non-surgical treatment-related assessment based on pre-SBRT CT and T1WI MRI features, and a combined model had an AUC of 0.878. Chen et al. [72] developed an MRI-based radiomics model based on pre-treatment MRI to predict local progression after SBRT, and the combined model achieved an AUC of 0.828. Candito et al. [74] developed an automatic analysis program based on WB-DWI to quantify the range of whole-body bone metastasis and evaluate the effect of treatment. The software had an accuracy of 80.5% for the assessment of treatment response and took about 90 s per case for analysis. Patients with multiple metastases need manual delineation of numerous lesions, and it is difficult to determine how many lesions have changed before and after treatment. Therefore, this tool may reduce the workload.

The scope of MRI-AI applications now includes treatment risk assessment and response monitoring. It can predict the amount of bleeding during surgery to support better preoperative preparation, predict fracture and local progression after radiation therapy to help plan follow-up strategies, and use automated quantification of WB-DWI to provide objective imaging indicators of response to systemic therapy.

## 4 Discussion

**Fig. 3:**
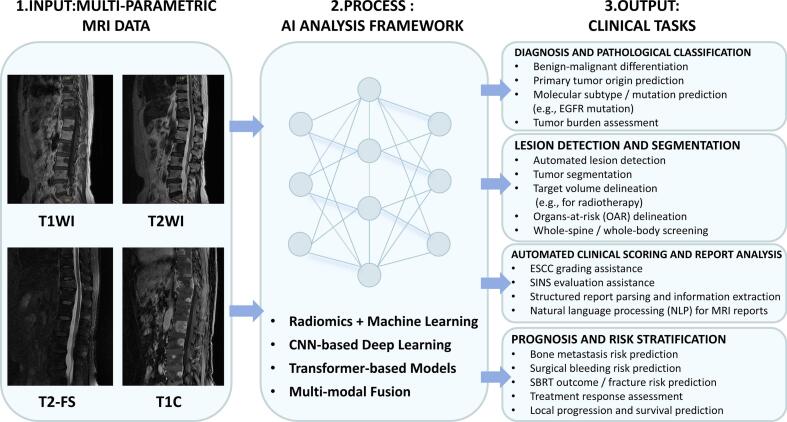
Conceptual framework of MRI-based artificial intelligence in spinal metastases, illustrating the workflow from multi-parametric MRI input and AI analysis framework to major clinical tasks.

This systematic review included 61 original studies and found that MRI-based AI technologies have been applied to many parts of the diagnosis and treatment of spinal metastases, such as differential diagnosis, lesion detection and segmentation, primary tumor prediction, gene mutation prediction, radiotherapy-related image processing, Bilsky grading, SINS-assisted calculation and treatment-related risk assessment (Fig. 3).

Most current research focuses on the diagnosis and differentiation of lesions and radiotherapy-related image processing. These studies have shown that MRI images contain general information about lesions, such as their shape, signal intensity and enhancement characteristics. More recently, quantitative features related to tumor origin, molecular characteristics, local aggressiveness and other prognostic indicators have also been identified. In short, the main purpose of MRI-AI is to extract relatively stable diagnostic indicators from complex imaging data, improve the efficiency of image assessment, reduce repeated manual delineation, and provide supplementary information for lesion quantification and comparison of treatment response.

At present, most studies still use image-task metrics such as AUC, accuracy and Dice coefficient as the main endpoints, while evidence directly related to clinical decision-making remains limited. Management of spinal metastases not only needs to determine whether metastatic lesions are present, but also requires assessment of the extent of spinal cord compression, spinal stability, treatment tolerance, post-radiotherapy fracture risk and expected survival. Existing studies on Bilsky grading, SINS scoring, intraoperative massive blood loss prediction, vertebral compression fracture after SBRT, and local progression prediction have begun to approach the problems of clinical management [53], [54], [55], [56], [57], [71], [73], [72], [74]. However, the number of related studies remains small, and there is still no direct evidence showing that these models can truly change treatment plans or improve patient outcomes.

Another issue is that the study groups are not evenly distributed. Most current studies are on prostate cancer and lung cancer. Most studies on prostate cancer have focused on predicting the risk of bone metastasis, segmenting pelvic metastases, and WB-DWI quantification of whole-body bone metastatic burden [58], [59], [60], [61], [62], [63], [64], [74]. Most studies on lung cancer have focused on predicting the primary tumor and EGFR mutation status [29], [30], [31], [32], [33], [34], [35], [36]. Cancer-specific models are not suitable for other frequent origins of spinal metastases, such as breast cancer, kidney cancer, thyroid cancer and gastrointestinal tumors. Primary tumors have different patterns of bone destruction, vascularity, treatment response and survival prognosis. Therefore, models built only on a few types of cancer may not be directly applicable to all patients with spinal metastases.

Prognostic prediction and monitoring of long-term treatment response are also still deficient. Most treatment decisions for spinal metastases are based on predicted overall survival, and MRI-AI models for overall survival or progression-free survival remain scarce. Among other applications of deep learning in survival analysis based on various types of data, such as images, text, and omics [75], most current research on spinal metastases has focused more on diagnosis, segmentation, and short-term risk prediction. In the future, some studies may show that certain patients are more likely to respond positively to surgery, radiotherapy or systemic treatment depending on the characteristics of their tumors and other reasons.

Generalization and interpretability of the model are also required for clinical application. Most studies were single-center and retrospective, and had small sample sizes. MRI sequences, scanning parameters, lesion annotation methods and evaluation criteria all differed among the studies. Previous studies have shown that medical AI models are sensitive to changes in data distribution, and thus their performance may decline due to differences between the training and testing datasets [76]. Therefore, future research should focus on strengthening multicenter external validation, prospective studies and standardized modeling workflows. At the same time, AI output is more suitable for alerting, initial screening, assisted scoring and risk stratification, but not for replacing clinicians in final decision-making.

In short, MRI-based AI technology has shown some application prospects for the diagnosis and treatment of spinal metastases in the following areas: differential diagnosis, lesion detection and segmentation, automated scoring, and treatment-related risk assessment. However, most of the current evidence is still based on technical performance verification, and there are no studies that directly investigate treatment selection, survival prognosis and long-term treatment response monitoring. In the future, the aim should be to develop a clinically feasible model rather than only a high-accuracy one. Specifically, problems such as a lack of external validation, uneven coverage of different cancer types, poor multimodal integration, and limited model interpretability need to be addressed.

## CRediT authorship contribution statement

**Chunhua Hou:** Writing – original draft, Methodology, Investigation, Conceptualization. **Anqi Wang:** Visualization, Validation, Data curation. **Jianru Xiao:** Writing – review & editing, Supervision, Resources, Project administration. **Xiang Wang:** Writing – review & editing, Visualization, Formal analysis, Data curation. **Wei Xu:** Writing – review & editing, Visualization, Supervision, Project administration, Conceptualization.

## Declaration of competing interest

The authors declare that they have no known competing financial interests or personal relationships that could have appeared to influence the work reported in this paper.

## Appendix A Supplementary data

Supplementary material 1 Supplementary Table S1. Glossary of AI, MRI and Clinical Terms in Spinal Metastases.

Supplementary material 2 Supplementary Table S2. Evaluation Metrics in MRI-based AI Studies.
