# Supplementary material for: Clinical applications of MRI-based artificial intelligence in spinal metastases: A systematic review
Source: J Bone Oncol. 2026 Jul 6;59:100782. doi: 10.1016/j.jbo.2026.100782 (PMC13356643; doi:10.1016/j.jbo.2026.100782)
Supplement: Supplementary material 1 — Supplementary Table S1. Glossary of AI, MRI and Clinical Terms in Spinal Metastases. [file mmc1.docx]

Supplementary Table S1

Glossary of AI, MRI and Clinical Terms in Spinal Metastases

| Term | Full name | Definition / Explanation in spinal metastases MRI-AI context |
| --- | --- | --- |
| CNN | Convolutional Neural Network | Deep learning model for extracting imaging features from MRI. |
| ResNet-50 | Residual Network-50 | Deep CNN for stable and accurate image classification. |
| U-Net | U-shaped Network | Standard model for medical image segmentation. |
| 3D U-Net | 3D U-shaped Network | Extension of U-Net for volumetric MRI segmentation. |
| Siamese Network | Siamese Neural Network | Model for comparing paired images for similarity learning. |
| Transformer | Transformer Model | Attention-based model for global feature extraction in MRI. |
| CNN-Transformer | CNN-Transformer Hybrid Model | Combines CNN and Transformer for local and global feature fusion. |
| Radiomics | Radiomics Analysis | Extraction of quantitative imaging features for prediction models. |
| ML | Machine Learning | Classical algorithms using handcrafted imaging features. |
| DL | Deep Learning | End-to-end neural networks for automatic feature learning. |
| DWI | Diffusion-weighted imaging | MRI sequence reflecting tumor cellularity and diffusion. |
| DCE-MRI | Dynamic contrast-enhanced MRI | MRI sequence assessing tumor perfusion and vascularity. |
| T1WI | T1-weighted imaging | MRI sequence for anatomy and bone marrow evaluation. |
| T2WI | T2-weighted imaging | MRI sequence sensitive to edema and soft tissue changes. |
| T2-FS | Fat-suppressed T2-weighted Imaging | Fat suppression MRI for better lesion visualization. |
| STIR | Short Tau Inversion Recovery | Strong fat suppression sequence for bone metastasis detection. |
| WB-DWI | Whole-Body Diffusion-Weighted Imaging | Whole-body MRI for metastatic burden assessment. |
| pseudo-CT | Synthetic CT (pseudo-CT) | CT-like images generated from MRI using deep learning. |
| GAN | Generative Adversarial Network | Deep learning model for image synthesis and enhancement. |
| LLM | Large Language Model | NLP model for extracting structured MRI report information. |
| SINS | Spinal Instability Neoplastic Score | Clinical score for evaluating spinal stability in metastases. |
| MESCC | Metastatic Epidural Spinal Cord Compression | Tumor-induced spinal cord compression. |
| Bilsky Grading | Bilsky Epidural Compression Grading | MRI-based grading system for spinal cord compression severity. |
| End-to-end DL model | End-to-end Deep Learning Model | Model that learns directly from MRI images without manual feature extraction. |
| Deep learning denoising reconstruction | Deep Learning Denoising Reconstruction | DL method used to reduce image noise and improve MRI quality. |
| Denoised DWI | Denoised Diffusion-weighted Imaging | DWI images processed to reduce noise and improve lesion visibility. |
| DL-generated pseudo-CT | Deep Learning-generated Synthetic CT | CT-like images generated from MRI for radiotherapy planning. |
| cGAN | Conditional Generative Adversarial Network | GAN model that generates images using specific input conditions. |
| GAN-based multimodal fusion model | Generative Adversarial Network-based Multimodal Fusion Model | Model that combines information from different imaging modalities. |
| Few-shot learning | Few-shot Learning | Learning method using only a small number of examples. |
| In-context learning | In-context Learning | LLM method that uses examples in the prompt to complete a task. |
| LLM-assisted scoring | Large Language Model-assisted Scoring | Use of LLMs to help calculate clinical scores from reports. |
