# Supplementary material for: Clinical applications of MRI-based artificial intelligence in spinal metastases: A systematic review
Source: J Bone Oncol. 2026 Jul 6;59:100782. doi: 10.1016/j.jbo.2026.100782 (PMC13356643; doi:10.1016/j.jbo.2026.100782)
Supplement: Supplementary material 2 — Supplementary Table S2. Evaluation Metrics in MRI-based AI Studies. [file mmc2.docx]

Supplementary Table S2

Evaluation Metrics in MRI-based AI Studies

| Term | Full name | Definition / Explanation in spinal metastases MRI-AI context |
| --- | --- | --- |
| DSC | Dice Similarity Coefficient | Measures overlap in segmentation tasks. |
| AUC | Area Under ROC Curve | Measures overall classification ability across thresholds. |
| ICC | Intraclass Correlation Coefficient | Metric measuring agreement in scoring systems. |
| ACC | Accuracy | Proportion of correctly classified samples. |
| Correct Classification Rate | Correct Classification Rate | Percentage of correctly classified cases in classification tasks. |
| Balanced Accuracy | Balanced Accuracy | Average of sensitivity and specificity, used for imbalanced data. |
